# *Pneumocystis jiroveci* pneumonia, *Nocardia brasiliensis,* and *Mycobacterium tuberculosis* co-infection in a myasthenia gravis patient: A case report

**DOI:** 10.1097/MD.0000000000024245

**Published:** 2021-01-08

**Authors:** Jiahui Hou, Junmin Cao, Panli Tan, Ying Yu

**Affiliations:** Department of Laboratory Medicine, The First Affiliated Hospital of Zhejiang Chinese Medical University, Hangzhou, China.

**Keywords:** co-infection, *Mycobacterium tuberculosis*, *Nocardia brasiliensis*, *pneumocystis jiroveci* pneumonia

## Abstract

**Rationale:**

Myasthenia gravis (MG) is an autoimmune disorder of the neuromuscular junctions that leads to fluctuating weakness and disabling fatigability. Due to difficulty in breathing caused by weakness of the respiratory muscles, patients with MG are more susceptible to pneumonia and other respiratory infections. As many patients with MG are given immunosuppressive therapy, this makes them more prone to infections. However, coinfection with 3 pathogens is very rare.

**Patient concerns:**

Here, we report the case of a 41-year-old gentleman with MG who was receiving long-term steroid therapy. He presented with a cough with pale brown expectoration that occurred without obvious inducement, severe pain in the scapula, as well as swelling and weakness of both legs. Despite undergoing treatment, but his symptoms did not improve, prompting two additional hospital admissions over a period of several months.

**Diagnosis:**

Bronchoscopy and bronchoalveolar lavage (BAL) were performed, revealing the presence of *Pneumocystis jirovecii* , *Nocardia brasiliensis,* and *Mycobacterium tuberculosis* (MTB). *N brasiliensis* was identified by positive modified acid-fast Kinyoun staining as well as a positive colony culture identified by matrix-assisted laser desorption ionization-time of flight mass spectrometry from the BAL sample. MTB was confirmed using GeneXpert, and due to the limitations of the culture conditions, methenamine silver stain was used to confirm *Pneumocystis jirovecii*. Next-generation sequencing (NGS) assay of the BAL samples also confirmed these pathogens.

**Interventions:**

The patient was transferred to a designated tuberculosis hospital and received anti-infective and anti-TB treatment.

**Outcomes:**

During treatment at the designated hospital, the patient developed gastrointestinal bleeding and impaired liver function. One month later, he developed multiple organ failure, consolidation of the left lower lung, and pan-drug resistant bacteremia. He refused further treatment and was discharged

**Conclusion:**

In conclusion, physicians should be aware of the predisposition of MG patients to co-infections, especially patients with metabolic disorders, to avoid inadequate treatment and poor patient outcomes. Due to the limitations of culture conditions, NGS should be considered as a new technique for identifying pathogens.

## 1 Introduction

*Pneumocystis jirovecii*, which causes pneumonia, and *Mycobacterium tuberculosis* are the most commonly identified infections in patients with myasthenia gravis (MG).^[1,2]^*Nocardia* species are potential opportunistic pathogens that are prevalent in people with compromised cell-mediated immunity. However, concurrent infection with these two pathogens is a rare event in immunocompromised individuals. The rarity of concurrent infection leads to the possibility that the detection of one pathogen may lead to low suspicion of the presence of a second infection. Thus, this may lead to inadequate treatment and poor patient outcomes. Additionally, since patients with co-infections are mostly immunocompromised individuals, an overlooked diagnosis can worsen the clinical outcome. Herein, we present an unusual case of a patient with MG who was receiving long-term steroid therapy combined with additional antimetabolite immunosuppressives and developed co-infections with *Pneumocystis jiroveci*, *N brasiliensis,* and *Mycobacterium tuberculosis*.

## 2 Case description

### 2.1 Ethics statement

According to the hospital protocol, no formal ethics approval was required for this study. The patient agreed and provided written informed consent for publication of this report and any accompanying images.

### 2.2 Case introduction

**Figure 1:**
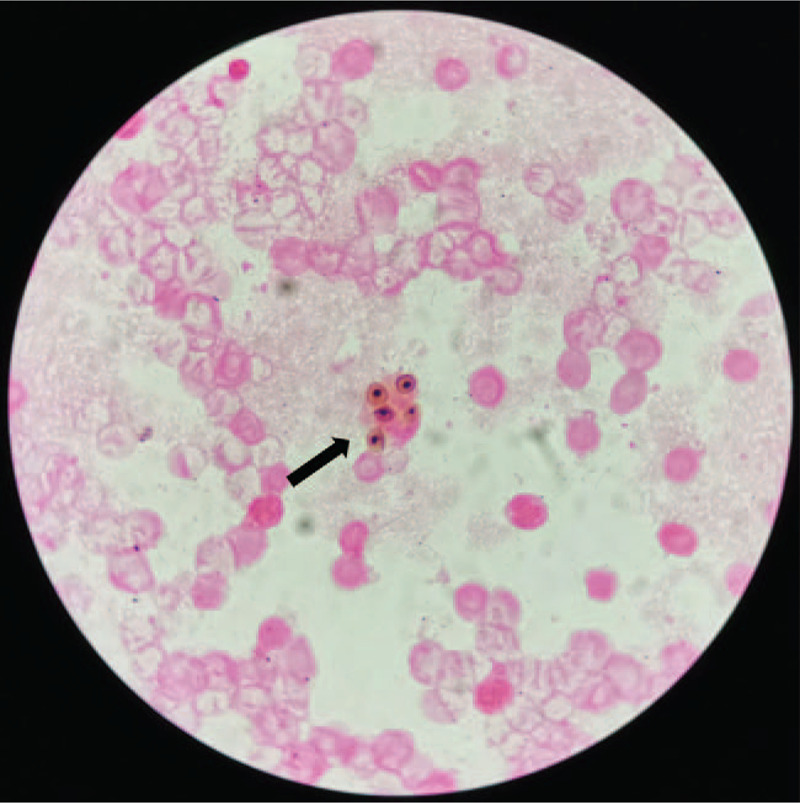
Sliver stain positive, original magnification × 40.

A 41-year-old man with a past medical history of type 2 diabetes mellitus and hyperlipidemia had been diagnosed with MG and underwent a surgical thoracotomy for thymoma 4 years ago. He was subsequently started on methylprednisolone and also received intravenous immunoglobulin. In August 2018, he was admitted to the hospital with respiratory obstruction and discharged after symptom alleviation. The symptoms recurred one month later, but they were again relieved on symptomatic treatment; thus, the patient was again discharged. Nevertheless, the symptoms persisted, prompting two additional hospital admissions over a period of several months. In November 2018, he was re-admitted with a cough with pale brown expectoration that occurred with no obvious inducement. This was accompanied by severe pain in the scapula and swelling of and weakness in both legs. The patient was initially treated with methylprednisolone 24 mg daily, which was later increased to 120 mg after re-admission. Despite these interventions, his symptoms persisted; 3 days after admission, the patient developed dyspnea and respiratory distress, with the maintenance of an upright posture required for breathing. His oxygen saturation was 75%, and he was transferred to the intensive care unit (ICU) for further treatment. He was started on broad-spectrum antibiotics including Sulperazon and voriconazole. Bedside chest radiographs showed diffuse hyperdense shadows in both lungs. Bronchoscopy was performed to rule out atypical infections such as *P jiroveci*. In addition, bacterial cultures, fungal cultures, and acid-fast bacilli cultures were performed on the bronchoalveolar lavage (BAL) samples, which were also delivered to the Beijing Genomics Institution (BGI) for next-generation sequencing (NGS). During this period, a prior BAL culture was analyzed, using the Zeihl-Neelsen acid-fast stain and the modified acid-fast Kinyoun stain, and showed positivity for *Mycobacterium tuberculosis and N brasiliensis*. Matrix-assisted laser desorption ionization-time of flight mass spectrometry (MALDI-TOF MS) of the colony culture identified the pathogen as *N brasiliensis*. Silver stain was positive for budding yeast (Fig. 1), which was identified as *P jiroveci*. Several days later, these results were confirmed by NGS. Subsequently, the patient was transferred to a designated tuberculosis hospital.

During hospitalization, the patient was treated with methylprednisolone 40 mg daily, anti-bacterial therapy (injections of sulfamethoxazole, Sulperazon, and linezolid), anti-fungal therapy (injections of voriconazole), and anti-TB therapy, including isoniazid, rifampicin, pyrazinamide, and ethambutol. On hospital day 15, the patient developed gastrointestinal hemorrhage and liver dysfunction. Therefore, the treatment was combined with liver-protecting agents and hemostatic therapy. On hospital day 23, he developed fever and shortness of breath. A chest computed tomography (CT) scan demonstrated consolidation of the left lower lung, and bacteremia caused by a multi-drug resistant *Acinetobacter baumanii* was diagnosed based on a positive blood culture. Consequently, the antibacterial agent was changed to tigecycline injection. However, the treatment was not effective. The patient refused further treatment and was discharged due to further clinical deterioration, including multiple organ failure, on hospital day 30.

## 3 Discussion

Although MG is a relatively rare condition, several patients with MG receive immunosuppressive therapy, with corticosteroids being the most commonly used immunosuppressive drugs. Tindall et al suggested that corticosteroids may reduce both the serum AChR antibody levels and the AChR reactivity of peripheral blood lymphocytes.^[3]^ Corticosteroids can inhibit the effects of lymphocytes, monocyte chemotaxis, and peripheral monocytes, including their bactericidal activity and the production of interleukin-1 and tumor necrosis factor-α. This can interfere with cell-mediated immunity, resulting in reduced host resistance to infections.^[4]^ The symptoms of MG can be aggravated by stress, surgery, various autoimmune or rheumatological diseases, thyroid dysfunction, and infectious diseases. In addition, systemic corticosteroids, especially when combined with other antimetabolite immunosuppressives, may increase the risk of opportunistic infections.

Previous studies have shown an association between the occurrence of TB and use of corticosteroids in some diseases that require steroid treatment.^[5,6]^ TB may arise by transmission from actively infected individuals or by the reactivation of a quiescent focus. As many patients with MG require immunosuppressive therapy, they are highly susceptible to contracting TB. Pulmonary TB can also exacerbate MG because of the respiratory distress in patients with MG. Some case reports have shown that concurrent TB can cause acute deterioration of MG.^[7]^ Ou et al analyzed the risk factors for TB in patients with MG by univariate Cox regression analysis and revealed that the risk factors are: age ≥ 60 years, presence of chronic obstructive pulmonary disease and/or underlying malignancy, use of corticosteroids, and use of high-dose pyridostigmine.^[2]^

*P jiroveci* pneumonia (PJP) is an opportunistic infection with a high mortality rate that is seen in patients receiving immunosuppressive therapy. *P jiroveci* lives almost exclusively in the pulmonary alveoli and adheres to the alveolar epithelium. Intra-alveolar macrophages serve as the primary host defense against *P jiroveci*, and macrophage deficiency or dysfunction can lead to infection.^[8]^ The most common underlying rheumatologic conditions associated with PJP are inflammatory myopathy, systemic lupus erythematosus (SLE), and granulomatosis with polyangiitis (GPA). A commonly cited recommendation based mainly on retrospective studies is to consider PJP prophylaxis in patients who are on ≥20 mg prednisone for ≥2 to 4 weeks.^[7,9]^

Pulmonary nocardiosis is a severe and uncommon opportunistic infection caused by *Nocardia* species. As potential opportunistic pathogens, *Nocardia* species are prevalent in individuals with compromised cell-mediated immunity. *Nocardia* does not belong to the normal human flora and is seldom a contaminant in tissue cultures.^[10]^ It is found in soil, decaying plants, and dust particles.^[11]^ The immunocompetence of the host determines the rate and course of infection. High-dose prolonged corticosteroid therapy is a known independent risk factor for infection with *Nocardia* because it suppresses Th1 cellular immunity.^[10]^

On conducting a literature review with the articles retrieved using the search terms *P jiroveci* pneumonia, *N brasiliensis,* and *Mycobacterium tuberculosis* on PubMed, there were some case reports on the co-existence of TB and *P jiroveci* pneumonia in MG patients, but co-infection with *P jiroveci*, *N brasiliensis,* and *Mycobacterium tuberculosis* in MG patients was rarely mentioned. In our case, the patient had a past medical history of type 2 diabetes mellitus and hyperlipidemia and had recently received immunosuppressive therapy with a high dose of methylprednisolone for four years. Additionally, he had undergone surgical thoracotomy for thymoma, further increasing the risk of opportunistic infections. It is essential to highlight this case given that most medical practitioners frequently manage patients with immune-compromised states, such as those on chronic steroid therapy. Identification of each of the underlying causative organisms is essential because this has significant implications for treatment. The application of new techniques for diagnosing pathogens, such as NGS, should be considered, particularly when the culture conditions are limited. Since co-infection is rare, when one infection is identified, most health professionals will have a low suspicion for an additional co-infection. This may lead to inadequate treatment and poor patient outcome. Hence, this case report is important since it can aid in increasing awareness among general practitioners.

## Author contributions

**Conceptualization:** Jiahui Hou.

**Methodology:** Junmin Cao.

**Validation:** Ying Yu.

**Writing – original draft:** Jiahui Hou.

**Writing – review & editing:** Panli Tan.
